# Genomic Analysis Unveils the Pervasiveness and Diversity of Prophages Infecting *Erwinia* Species

**DOI:** 10.3390/pathogens12010044

**Published:** 2022-12-27

**Authors:** Tulio Morgan, Rafael Reis de Rezende, Thamylles Thuany Mayrink Lima, Flávia de Oliveira Souza, Poliane Alfenas-Zerbini

**Affiliations:** Laboratório de Vírus, Departamento de Microbiologia, Instituto de Biotecnologia Aplicada à Agropecuária, Universidade Federal de Viçosa, Viçosa 36570-900, MG, Brazil

**Keywords:** *Erwinia*, prophage, *Caudoviricetes*, sequence diversity, anti-phage defense systems

## Abstract

Prophages are abundant elements integrated into bacterial genomes and contribute to inter-strain genetic variability and, in some cases, modulate the environmental behavior of bacteria, such as pathogen virulence. Here, we described prophage occurrence and diversity in publicly available *Erwinia* genome assemblies, a genus containing plant pathogens. Prophage-like sequences were identified and taxonomically classified. Sequence diversity was analyzed through intergenomic similarities. Furthermore, we searched for anti-phage defense systems in *Erwinia* spp., such as DISARM, BREX, and CRISPR-Cas systems, and identified the putative targets of CRISPR spacers. We identified 939 prophage-like sequences in 221 *Erwinia* spp. genome assemblies. Only 243 prophage-like sequences were classified, all belonging to the *Caudoviricetes* class. The set of putative *Erwinia* prophages was mostly unique since only three sequences showed more than 70% intergenomic similarities to known *Erwinia* phages. Overall, the number and type of CRISPR-Cas systems were conserved within *Erwinia* species, with many spacers directed to the putative prophages identified. This study increased the knowledge of the diversity and distribution of *Erwinia* prophages, contributing to the characterization of genetic and ecological factors influencing *Erwinia* spp. environmental fitness.

## 1. Introduction

The *Erwinia* genus (family: *Erwiniaceae*, order: *Enterobacteriales*, class: *Gammaproteobacteria*) comprises a heterogeneous group of bacteria recognized for its pathogenicity against a wide range of plants, including crops from *Rosaceae*, *Myrtaceae,* and *Cucurbitaceae*. *Erwinia amylovora* was the first bacterium identified as causing disease in plants, with bacterial colonization generally beginning in the flowers or shoot tips, followed by migration into plant tissues [1]. Currently, many *Erwinia* species are described as economically important plant pathogens, including *E. pyrifoliae* [2], *E. tracheiphila* [3], and *E. psidii* [4]. The management of *Erwinia* disease in plants includes the use of antibiotics, such as streptomycin, but the emergence of bacterial resistance [5] and policies to restrict its use makes necessary the development of alternative control strategies, such as bacteriophage control [6,7].

Bacteriophages (phages) are ubiquitous in natural environments and are powerful drivers of bacterial evolution, such as providing antibiotic-resistance genes, virulence-related genes, and resistance to lytic bacteriophages [8]. Besides shaping cell fitness when integrated into bacterial chromosomes, prophages may shift from a lysogenic to a lytic life cycle depending on the environmental conditions, causing cell lysis and a shrinkage in the bacterial population [9]. The cell lysis promoted by most phages is highly selective, making them appealing to biocontrol pathogenic bacteria since other microbes are virtually unaffected.

Besides the relevance of phages to bacterial ecology and evolution, few studies investigating the diversity of (pro)phages of *Erwinia* were carried out using genomic data [10,11]. A comprehensive description of prophage occurrence may be helpful to deeply characterize *Erwinia* pathogenicity and the development of efficient control strategies, such as a better understanding of bacterial susceptibility to lytic phages and the evolution of antibiotic resistance genes.

Although biocontrol using phages is promising, bacteria can circumvent phage infection using a range of mechanisms, such as CRISPR-Cas [12], DISARM [13], and BREX [14] systems. The CRISPR-Cas system is an adaptive system based on nucleotide sequence complementarity between the CRISPR spacer and the target mobile genetic element (MGE), meaning that past contacts with phages and other MGEs are recorded to prevent future infections. DISARM and BREX systems are based on host DNA methylation followed by restriction–modification of a phage genome (DISARM) or blocking phage replication/integration (BREX). Thus, the description of these systems could provide clues on bacterial immunity to phages.

In this study, we aimed to broadly describe the *Erwinia* prophage distribution and diversity, including the identification of novel sequences. We used PHASTER to scan publicly available *Erwinia* genomes for prophage-like sequences. Taxonomical assignments and sequence diversity analysis indicated that nearly all identified sequences were unrelated to previously described *Erwinia* (pro)phages. Based on prophage profiles, we pointed out that their occurrence is highly dependent on the *Erwinia* species and, thus, mainly influenced by the species-specific genetic background. Furthermore, we searched for anti-phage defense systems in *Erwinia* genomes to acquire information on the bacterial susceptibility to known *Erwinia* phages and the putative prophages identified in this work.

The data generated here contribute to describing the interplay between phages and *Erwinia* and could serve as a starting point for future studies regarding *Erwinia* pathogenicity and environmental fitness.

## 2. Materials and Methods

### 2.1. Bacterial Genomic Sequences and Quality Assessment

From GenBank, 258 *Erwinia* spp. genome sequences were downloaded in April 2022 (search parameters: “Erwinia” [Organism] OR erwinia [All Fields]) AND (latest [filter] AND all [filter] NOT anomalous [filter]). To assess the genome completeness, we searched for 440 *Enterobacterales* universal single-copy orthologous genes using BUSCO v.5.0.0 [15] (parameters: -l enterobacterales_odb10 -m genome). Only the genomes with completeness equal to or greater than 95% were kept for the subsequent analysis.

The phylogenomic tree was built using the orthologous genes in *Erwinia* high-quality assemblies. First, the core genome was identified using Roary v.3.13.0 [16], considering that a “core gene” occurs in at least 99% of *Erwinia* genomes analyzed. The multiple sequence alignments were conducted using Mafft v.7.471 [17], and the phylogenetic reconstruction was carried out by Fasttree v.2.1.9 [18] using the GTR substitution model.

### 2.2. Identification of Prophage-like Sequences within Erwinia spp. Genomes

The *Erwinia* genome sequences were submitted to PHASTER [19] via the API to predict candidate prophage sequences. When needed, the submissions were adjusted to handle fragmented assemblies.

Since many *Erwinia* spp. genome assemblies were fragmented (had dozens of contigs), we analyzed if the incomplete or questionable sequences identified by PHASTER were near the contig edges. This may indicate that the prophage-like sequences could also be artificially fragmented during genome assembly, hindering the completeness estimated by PHASTER. Following this, the putative prophages occurring in 1000 bp or less from contig edges and sizes less than 20 kb were considered a possible artificially fragmented sequence.

All identified prophage-like sequences were submitted to Prokka v.1.14.5 [20] for open-reading frame prediction (parameters: —kingdom Viruses—gcode 11).

### 2.3. Taxonomic Classification of Putative ERWINIA Prophages

The taxonomic assignment of the candidate prophage sequences was conducted using vConTACT2 [21], a gene-sharing network-based system for viral classification. The input sequences had taxonomy assigned (family/genus) as long as they belonged to a cluster containing reference sequences (vConTACT2 parameters: —rel-mode ‘Diamond’—db ‘ProkaryoticViralRefSeq201-Merged’—pcs-mode MCL—vcs-mode ClusterONE).

Furthermore, we used VPF-class to attempt to improve the taxonomic classification of putative prophages. This tool compares phage-predicted proteins against Hidden Markov Models (HMMs) representing Viral Protein Families (VPFs), which are groups of viral orthologous proteins exploited in determining viral taxonomy and hosts. For each input contig, VPF-class calls Prodigal (v.2.6+) for gene finding, followed by alignments using hmmsearch (HMMER v.3.2+) between the predicted proteins and the set of classified VPFs. We assigned taxonomy to the putative prophages using thresholds of 0.8 for confidence score and 0.5 for membership ratio, which yielded satisfactory accuracy and recovery in tests on prophage sequences [22].

Finally, we used VIRIDIC [23] to obtain an overview of whole-sequence similarity between the candidate prophages. VIRIDIC uses local alignments (BLASTn) to calculate nucleotide-based intergenomic similarities and sequence clustering in “species_cluster” and “genus_cluster” as long as they share sufficient intergenomic similarities (default threshold of 95% for species and 70% for genus).

Given that viral taxonomy has been constantly updated by the International Committee on Taxonomy of Viruses (ICTV), the classification of algorithms may have a delay to accommodate the changes. Therefore, some discrepancies may occur between the viral sequences classified here and the current viral taxonomy (https://ictv.global/, accessed on 17 October 2022).

The bar plots were generated using the ggplot2 package [24] in the R environment (R Core Team, 2013).

### 2.4. CRISPR-Cas Systems Identification

The putative CRISPR-Cas systems were identified using CRISPRCasFinder v.4.2.20 [25] with default parameters. The predictions were filtered to keep highly likely CRISPR-Cas systems containing at least one Cas operon and CRISPR arrays with evidence levels 3 or 4.

### 2.5. Protospacer Identification

To identify the putative protospacers, the spacer sequences from the CRISPR arrays were submitted to BLASTn alignments using a database composed of plasmid (133,394 entries) and viral (532,025 entries) sequences obtained from GenBank [26] in October 2021, as well as 2,377,994 sequences of cultivated and uncultivated viruses from the IMG/VR database [27]. Furthermore, the database was updated in September 2022 to include 399 sequences from phages infecting *Erwinia* and other genera retrieved using the search parameters (“Erwinia” [Organism] OR erwinia [All Fields]) AND (viruses [filter] AND (“1000” [SLEN]: “500,000” [SLEN])). The local alignments were conducted with the following parameters: -word_size 7 -penalty -1 -reward 1 -gapopen 10 -gapextend 2 -evalue 10. Alignment hits having at least 95% of query coverage and identity were considered significant. The best-hit for each query (spacer) was assigned based on the following criteria by order of importance: (i) greater query coverage; (ii) greater alignment identity; (iii) sequences from GenBank. Stacked bar plots showing the frequency of putative protospacers were built using the ggplot2 package [24] in the R environment (R Core Team, 2013).

### 2.6. In Silico Screening for DISARM System in Erwinia spp. Genomes

The hmmscan (HMMER v.3.2+) was used to search the profile-HMMs of the three core proteins of the DISARM system (drmA (PF00271: Putative helicase domain), drmB (PF09369: DUF1998, helicase-associated domain) and drmC (PF13091: Phospholipase D/nuclease domain) [13]) against the predicted proteomes of *Erwinia* spp. The alignments were filtered using the cutoffs: e-value < 0.001, profile-HMM coverage > 35%, and score > 20. After that, a manual inspection was conducted to check the genomic positions of the three core genes in the *Erwinia* genomes and if they resemble the DISARM system structure [13].

### 2.7. In Silico Screening for BREX Types 1–6 in Erwinia spp. Genomes

The accession numbers of core proteins of BREX types 1 to 6 were obtained in [14], which included the PglZ and BrxC/PglY proteins. The sequences were downloaded from GenBank and subjected to BLASTp searches against the predicted proteomes of *Erwinia* spp. Only alignments with identity ≥40% and subject cover ≥50% were considered significant. After that, a manual inspection was conducted to check the genomic positions of the two core genes in the *Erwinia* genomes and if they resemble the BREX system structure [14].

## 3. Results

### 3.1. Acquisition of Erwinia spp. Genome Assemblies and Quality Assessment

To broadly describe the occurrence and diversity of putative *Erwinia* prophages, we screened all *Erwinia* spp. genome assemblies publicly available in the NCBI GenBank database (April 2022). First, we downloaded 258 *Erwinia* spp. genomic sequences and performed completeness quality filtering using BUSCO v5 to remove poorly assembled genomes. Using a completeness threshold of 95%, 221 assemblies were kept for the subsequent analysis, representing approximately 88% of the initial data set, spanning 19 *Erwinia* species (Figure S1A).

Most high-quality assemblies were from *Erwinia amylovora* (146 sequences). In contrast, many species, such as *E. psidii, E. mallotivora*, and *E. typographi*, were under-represented, showing only one high-quality genomic sequence each. In addition, a large proportion of the high-quality assemblies were from *Erwinia* sp., i.e., bacteria that were not yet classified at the species level (Figure S1A). *Erwinia* spp. are mainly found associated with plants, with the most common hosts from *Rosaceae* and *Brassicaceae* families. However, these bacteria were also found to be associated with invertebrates and one associated with a bird (Figure S1B). In addition, a few high-quality assemblies were obtained from metagenomic studies, comprising 6.3% of all *Erwinia* spp. genome assemblies (Figure S1C).

The source locations of *Erwinia* spp. were concentrated in the northern hemisphere, primarily due to the high number of *E. amylovora* sampled in North America (Figure S1D), where this species generally poses a higher threat to crop production, including apple and pear. Nevertheless, the occurrence of *Erwinia* species has been reported in countries below the equator, such as *E. psidii* in Brazil and *E. amylovora* in New Zealand [4,28]. Furthermore, the first high-quality assemblies of *Erwinia* spp. were deposited in databases in 2008, followed by a significant increase of sequences made available by 2020, comprising especially *E. amylovora* [28] (Figure S1E).

### 3.2. Prophage-like Sequences Are Pervasive in Erwinia spp. Genomes

**Figure 1:**
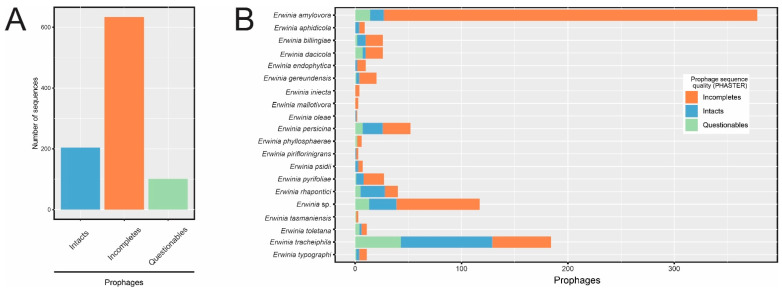
Candidate prophage sequences identified in *Erwinia* spp. genomes using PHASTER. (**A**) Number of prophage sequences according to the completeness level. (**B**) Breakdown of prophage sequences detected in each *Erwinia* species.

The 221 high-quality bacterial genome assemblies were subjected to a prophage sequence search using PHASTER [19]. A total of 939 prophage-like sequences were detected, most of which comprised incomplete sequences (Figure 1A), especially in *E. amylovora* assemblies (Figure 1B). Few isolates of this species showed intact or questionable sequences. In contrast, intact prophages were in higher proportion in some *Erwinia* species, such as *E. rhapontici* and *E. tracheiphila*.

We detected 67 incomplete and questionable prophage-like sequences (67/939 = 7.1%) near contig edges and had less than 20 kb (Table S1), indicating that they might be fragmented during the bacterial genome assembly process. Because of this, a prophage sequence fragmented between bacterial contigs may be considered as different prophages, and its completeness could be misassigned by PHASTER due to the non-contiguity of ORFs. Although showing signals of misassembly, we cannot guarantee that the 67 sequences were indeed disrupted prophage genomes. Since these sequences were clearly of prophage origin, we kept them in the data set to ensure the complete description of putative *Erwinia* prophages.

**Figure 2:**
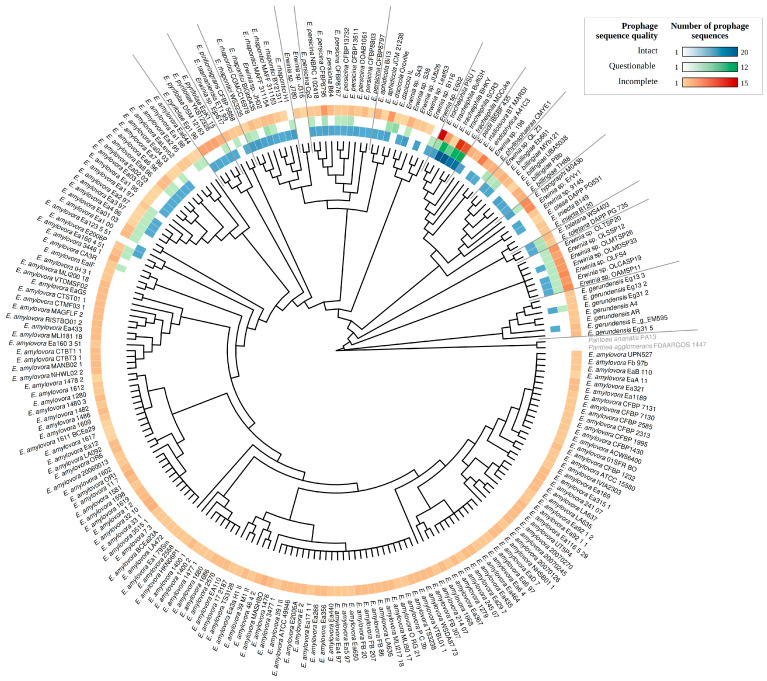
Phylogenomic analysis including 221 *Erwinia* spp. using 74 core genes (genes present in at least 99% of *Erwinia* genomes). The tree was built using Fasttree v.2.1.9, applying the GTR evolutionary model and computing local support values with the Shimodaira-Hasegawa test. The density color map next to the terminal nodes indicates the number of putative prophage sequences identified in each *Erwinia* genome using PHASTER. The tree was rooted at *Pantoea* species.

To ensure that the taxonomic classifications of the *Erwinia* spp. provided in GenBank were correct, and to better visualize the correlation between *Erwinia* classification and prophage occurrence, we reconstructed the phylogeny of the genus using 74 genes present in at least 99% of *Erwinia* genomes (core genes). The clades observed in the evolutionary model were consistent with the taxonomic classification given in GenBank (Figure 2). Furthermore, the putative prophages had a differential distribution within the *Erwinia* genus. While *E. amylovora* and *E. gerundensis* showed a lower prevalence of intact and questionable sequences, *E. tracheiphila* harbored a high density of putative prophages, including many intact sequences (Figure 2). All *Erwinia* spp. had genomic regions identified as prophage-like sequences, especially incomplete ones, which occurred in almost all assemblies analyzed (Table S2).

Considering only the intact sequences, polylysogeny was frequent in some *Erwinia* species, especially in *E. tracheiphila*, detecting up to 24 sequences in a single genome. On the other hand, we detected only one polylysogeny event in *E. amylovora* (strain E2006P), reflecting the scarcity of intact sequences in this species.

Bacteria and phages establish mutual evolution that is an important driver of these organisms’ ecological and evolutionary processes [29]. In bacteria-phage coevolution, many genome sequence-shaping events may occur, which include a convergence of %GC [30]. In this sense, we compared the GC content between the putative prophages and their hosts, and in most cases, the %GC was considerably divergent, showing a weak correlation (Figure S2).

### 3.3. Sequence Diversity and Taxonomic Classification of the Putative Erwinia Prophages

We used vConTACT2 to assess the evolutionary relatedness between the putative *Erwinia* prophages and viral RefSeq genomes. The vConTACT2 generates a network of virus genomes that are connected as long as they share proteins with sufficient sequence similarity, forming viral clusters (VCs).

**Figure 3:**
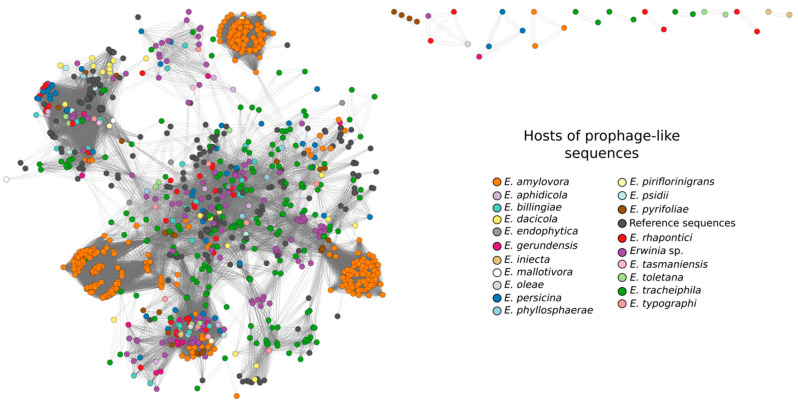
Shared gene content between the prophage-like sequences and reference phage genomes that showed connection to at least one of the putative prophages, as determined by vConTACT2. Network graph visualization included 920 clustered prophage-like sequences colored according to their hosts and 191 clustered reference phage genomes (dark gray nodes). Darker colors were more abundant in the network and belonged to hosts (*Erwinia* species) with more prophage-like sequences. The network was visualized in CytoScape using Edge-weighted Spring Embedded layout.

Overall, the nodes in the gene-sharing network were highly connected, reflecting that most putative prophages shared multiple genes with other sequences (Figure 3), regardless of their completeness (incomplete/questionable/intact). In general, the putative prophages from *E. amylovora* were clustered in three well-defined groups in the network due to the high similarity between these sequences. Only 29 putative prophages were not connected with the bulk network, representing more divergent sequences.

**Figure 4:**
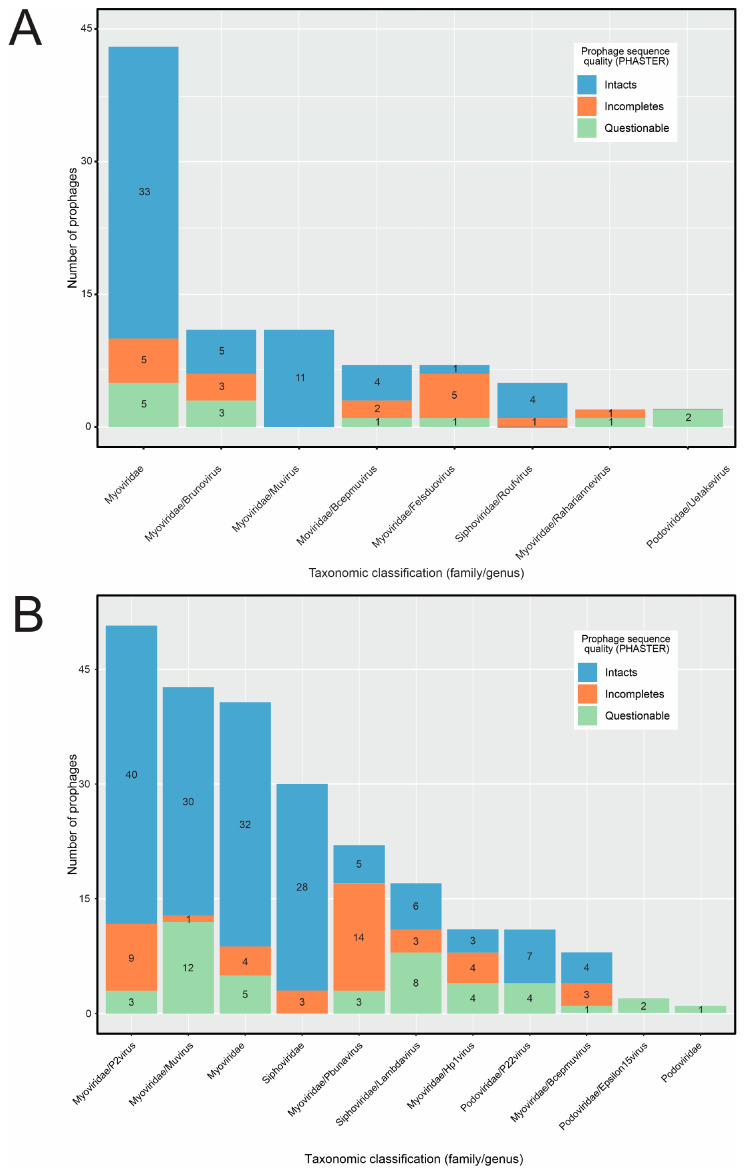
Taxonomic assignment of the putative *Erwinia* prophage sequences at family and genus levels. Sequences falling in each taxonomy were subdivided based on the completeness given by PHASTER. (**A**) Taxonomic classification provided by vConTACT2. The prophage sequences within viral clusters containing at least one sequence from databases (ProkaryoticViralRefSeq201-Merged) were classified based on the classification of the reference sequence. (**B**) Taxonomic classification provided by VPF-class. The taxonomy of the prophage sequences was assigned using thresholds of 0.8 for the confidence score and 0.5 for the membership ratio.

Regarding taxonomic assignment, 776 putative *Erwinia* prophages were grouped in 107 distinct VCs yet only 10 contained RefSeq genomes, making it possible to assign taxonomy to 88 putative *Erwinia* prophages (Figure 4A, Table S3). All sequences classified using vConTACT2 were from the *Caudoviricetes* class, mostly from the *Myoviridae* family (81/88). Only five putative prophages were classified in *Siphoviridae* and two in *Podoviridae*, the latter found exclusively in *Erwinia dacicola* genomes. Although *E. amylovora* genomes were abundant in the data set, only three putative prophages from this species could be classified (*Myoviridae* family), all assigned as intact sequences by PHASTER. Thus, the classification of putative *E. amylovora* prophages by vConTACT2 was probably hampered by the defective nature of sequences found in *E. amylovora* assemblies.

Afterward, we used VPF-class, attempting to increase the set of classified prophage-like sequences. While the vConTACT2 strongly depends on the reference genomes to classify phages, VPF-class uses statistical models representing sets of orthologous viral proteins, which tends to perform better in phage genomes with higher sequence diversification and variable gene content. Indeed, we could classify 239 putative *Erwinia* prophages using VPF-class, representing 25.4% of the data set (239/939) and 2.7 times more sequences than vConTACT2. Similarly to vConTACT2, most of the putative prophages belonged to the *Myoviridae* family (177/239), followed by *Siphoviridae* (48/239) and *Podoviridae* (14/239) (Figure 4B). Among the classified sequences, 155 were intact and 43 were questionable. Although abundant in the data set, the incomplete sequences were the least classified by VPF-class.

The majority of the putative prophages classified by vConTACT2 (84/88 = 95.4%) were also classified by VPF-class (the four exceptions included prophage_6#Erwinia_sp_S38, prophage_2#Erwinia_endophytica_A41C3, prophage_2#Erwinia_sp_S43, prophage_6#Erwinia_billingiae_Pbb) and only one sequence (1/84 = 1.2%) did not agree at family level classification between the two tools (the prophage_3#Erwinia_billingiae_Eb661 was classified in the *Myoviridae* family by vConTACT2 but in the *Siphoviridae* family by VPF-class) (Table S4).

Notably, 696 putative *Erwinia* prophages (696/939 = 74.1%) were not assigned to any viral taxonomy using vConTACT2 and VPF-class. In this set of unclassified sequences, 650 were defective (incomplete or questionable), while 46 were intact. Although the incomplete sequences were the most abundant in the data set (634/939 = 67.5%), they were the least classified (42/243 = 17.3%), indicating that the sensitivity of vConTACT2 and VPF-class decreased when dealing with fragmented or partial virus genomes, a known caveat of classification algorithms [31]. Considering the intact sequences, only 22.5% (46/204) could not be classified based on known viruses, indicating that some putative functional prophages lack similar counterparts in sequence databases. Among these unclassified intact sequences, 32 clustered in 17 VCs by vConTACT2 (Table S5), some of these VCs containing sequences classified by VPF-class. Thirteen sequences overlapped between distinct VCs, while one sequence was an outlier.

Given that the PHASTER identified only candidate prophages from the *Caudoviricetes* class, we employed VIBRANT v.1.2.1 [32] to increase the reliability of the taxonomic diversity of *Erwinia* prophages since these two tools use different approaches to identify these sequences. The VIBRANT tool retrieved 469 prophage-like sequences, which were classified into the families *Myoviridae* (138 sequences), *Siphoviridae* (33 sequences), and *Podoviridae* (8 sequences) by a combination of VPF-class and vConTACT2. Thus, the classification of putative prophages identified by the two tools was similar. Furthermore, we wondered if tools directed at finding viruses from specific taxonomic sections could improve the description and classification of *Erwinia* prophages. For this purpose, we employed the pipeline “Inovirus_detector” developed by [33] to search for inovirus-like sequences (class: *Tubulavirales*) on the 221 *Erwinia* spp. assemblies. However, no such sequences were retrieved, indicating that all of the publicly available *Erwinia* genomes harbored putative prophages only from the *Caudoviricetes* class.

**Figure 5:**
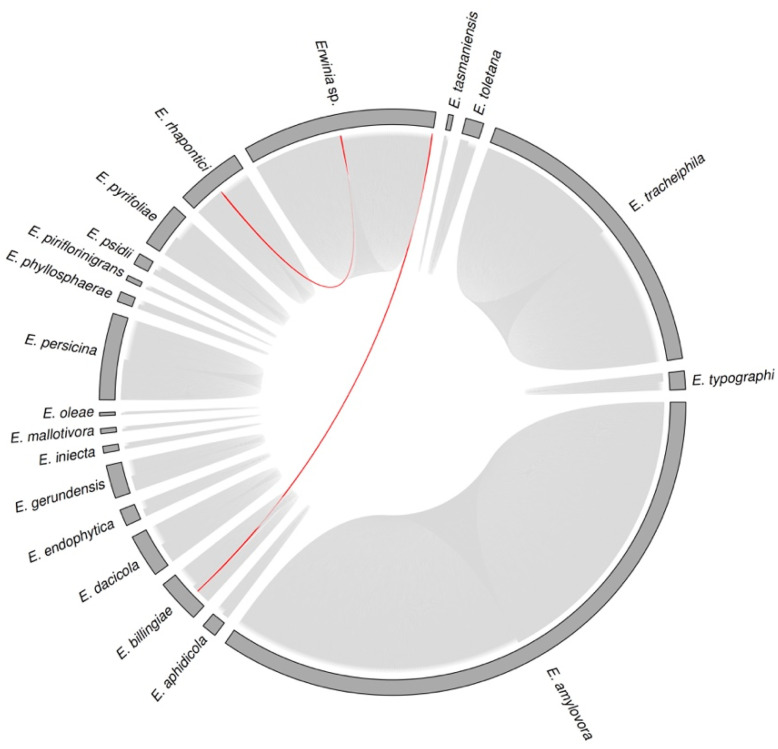
Diagram showing the hosts of each candidate *Erwinia* prophage identified by PHASTER (939 sequences). The sectors (grey bars) represent the *Erwinia* species. Each chord connecting the sectors represents a putative prophage. Prophages within a given “species cluster” (VIRIDIC) were considered the same virus species. Only two putative prophages of the same species occurred in different *Erwinia* spp. (red chords): the prophage_1#Erwinia_rhapontici_MAFF_311153 and prophage_4#Erwinia_sp_JH02 from “species cluster” 65; and the prophage_5#Erwinia_billingiae_Eb661, prophage_2#Erwinia_billingiae_MYb121, prophage_2#Erwinia_billingiae_UBA5038, and prophage_1#Erwinia_sp_QL_Z3 from “species cluster” 82 (Table S6).

Since most *Erwinia* prophage sequences were incomplete according to PHASTER, they may have many truncated or missing ORFs, hindering the taxonomic classification by vConTACT2 and VPF-class. So, we used VIRIDIC to improve the evolutionary analysis. VIRIDIC clustered the 939 sequences in 450 species clusters, implying that some putative prophages were highly similar (>95% of intergenomic similarity). The putative prophages of a given species cluster had a narrow host range, occurring in a specific *Erwinia* species (Figure 5). The only two apparent exceptions were the candidate prophages of *Erwinia* sp. JH02 (prophage_4#Erwinia_sp_JH02) and *Erwinia* sp. QL-Z3 (prophage_1#Erwinia_sp_QL_Z3), which occurred in the species cluster of *E. rhapontici* and *E. billingiae* prophages (Figure 5—red lines). In the phylogenomic model, the unclassified *Erwinia* isolates JH02 and QL-Z3 were in the clades of *E. rhapontici* and E. billingiae (Figure 2). As a result, such isolates could be classified as *Erwinia rhapontici* JH02 and *Erwinia billingiae* QL-Z3.

Regarding genus clusters, VIRIDIC generated 362 clusters of sequences that shared at least 70% of intergenomic similarities. As in species clusters, the putative prophage genera were host-specific, i.e., the prophage-like sequences within a given genus cluster occurred in a specific *Erwinia* species (Table S6).

Furthermore, only two known *Erwinia* phage sequences showed more than 70% of intergenomic similarities to the candidate prophages identified by PHASTER. The *Eganvirus EtG* (genus: *Eganvirus*, family: *Peduoviridae*, class: *Caudoviricetes*, https://ictv.global/taxonomy) was in the same genus cluster of prophage_5#Erwinia_tracheiphila_MDCuke and prophage_5#Erwinia_tracheiphila_SCR3, showing intergenomic similarities of 90.5% and 92.0%. The *Eganvirus EtG* is probably a temperate phage that infects many *E. tracheiphila* isolates [34]. Thus, prophage_5#Erwinia_tracheiphila_MDCuke and prophage_5#Erwinia_tracheiphila_SCR3 might also be active temperate phages since they were classified as intact by PHASTER. Likewise, the prophage_39#Erwinia_tracheiphila_MDCuke shared 98.5% of intergenomic similarity to the *Erwinia* phage LS-2018a (CP013974.1), a putative temperate phage sequenced together with *E. tracheiphila* MDcuke infecting *Cucumis melo* [3]. Thus, the prophage_39#Erwinia_tracheiphila_MDCuke was the same phage species detected in the *E. tracheiphila*, although it was classified as questionable by PHASTER.

### 3.4. CRISPR-Cas Systems Are Unevenly Distributed across the Erwinia Genus and Have a High Proportion of Unknown Targets

Among the 221 assemblies analyzed, 170 (76.9%) harbored putative CRISPR-Cas systems composed of at least one *Cas* operon and CRISPR arrays of evidence levels 3 or 4 (Table S7).

The CRISPR-Cas systems were unevenly distributed in the *Erwinia* genus, occurring in all *E. amylovora* and *E. pyrifoliae* strains, while they were completely absent in most other *Erwinia* species, such as *E. billingiae*, *E. gerundensis*, *E. rhapontici* and *E. tracheiphila*. Nevertheless, the absence of CRISPR-Cas systems in some species must be viewed with caution due to the low number of assemblies in the data set, providing an unreliable proxy of CRISPR-Cas system distribution.

Despite showing a high frequency in some species, the diversity of system types was narrow since only types I-E and I-F were detected. Furthermore, the presence and type of CRISPR-Cas systems in *Erwinia* were likely species-dependent, such as all *E. amylovora* assemblies containing type I-E and *E. pyrifoliae* assemblies containing type I-E and I-F. Regarding the CRISPR spacers, *E. amylovora* had the greatest variation in the number of arrays, ranging from two to eight. However, the average number of arrays was similar to that found in other *Erwinia* species, indicating that strains with higher content of CRISPR arrays were outliers (Table S7).

Furthermore, *Erwinia pyrifoliae* was the only species that harbored two different types of CRISPR-Cas systems. Although showing a high potential to prevent virus infection, a minimum of six putative prophages were detected in the *E. pyrifoliae* strains, each containing at least one intact sequence.

**Figure 6:**
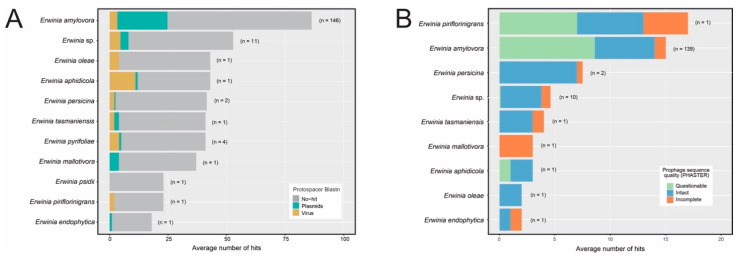
Average number of BLASTn hits between *Erwinia* CRISPR spacers and plasmids and virus sequences from databases (GenBank and IMG/VR v3). Significative BLASTn alignments had query (spacer) cover greater than 95% and identity greater than 95%. Only the best hit per query was taken. The number in parenthesis next to each bar indicates the number of genomes that harbored CRISPR-Cas systems containing at least one Cas operon and one CRISPR array with evidence level 3 or 4 according to the CRISPRCasFinder tool. (**A**) Alignments between CRISPR spacers and plasmids/virus sequences from databases. (**B**) Alignments between CRISPR spacers and prophage-like sequences detected in *Erwinia* genome assemblies using PHASTER.

The CRISPR arrays of the *Erwinia* spp. were analyzed to identify the putative targets of each spacer using BLASTn alignments against plasmid and viral sequences from GenBank, as well as viral sequences from IMG/VR v3 database. It is worth mentioning that the database contained an up-to-date set of *Erwinia* phage sequences available in GenBank (September 2022), including the 60 sequences from *Erwiniaceae* phages described in [10]. Most spacers did not align significantly to viral or plasmid sequences (Figure 6A), possibly due to sequence mutation or the absence of the target protospacers in databases. Regarding the spacers showing significant BLASTn hits, most of the *E. amylovora* and *E. pyrifoliae* spacers targeted plasmids. On the other hand, *E. aphidicola*, *E. oleae,* and *E. tasmaniensis* spacers were directed mainly to viruses. However, this may not represent the true diversity of protospacers in these species since only one high-quality genomic sequence of each one was publicly available for analysis.

Furthermore, we analyzed if the CRISPR spacers could target the candidate prophage sequences identified by PHASTER. Most *Erwinia* species with CRISPR-Cas systems had spacers targeting the putative prophages (157/170 = 92.4%), predominantly intact and questionable sequences (Figure 6B). This is evident for *E. amylovora*, where 139 assemblies (139/146 = 95.2% of the analyzed genomes) contained CRISPR spacers directed to the putative prophages, mainly against *E. amylovora* prophages occurring in different genomes (Table S8). On the other hand, although the four isolates of *Erwinia pyrifoliae* had CRISPR-Cas systems (Table S7), none of their spacers showed significant alignments to the putative prophage sequences.

### 3.5. BREX and DISARM Anti-Phage Defense Systems Are Rare in Erwinia spp. Genomes

Among the two hundred and twenty-one assemblies analyzed, only six (2.71%) harbored putative core genes of the BREX defense system. The *Erwinia* sp. Ejp617, *E. gerundensis* AR, and all four *E. pyrifoliae* isolates analyzed had the *pglZ* and *brxC* core genes separated by an additional gene, resembling the BREX type 1 system [14]. Further BLASTp alignments confirmed that the neighbor genes were the other ones that comprise a putative complete BREX system in these *Erwinia* isolates (Table S9).

On the other hand, none of the *Erwinia* genomes showed all three core genes from the DISARM system organized in a putative operon.

## 4. Discussion

In this study, we analyzed the occurrence and diversity of prophage-like sequences in 221 publicly available *Erwinia* genome assemblies. Over the years, there has been an increasing number of high-quality *Erwinia* genome assemblies deposited in public databases, reflecting the growth of interest in genomic characterization of this genus due to its relevance to crop production [35,36]. Many *Erwinia* species are associated with plant disease [37], to which prophages could contribute relevant genetic features such as virulence genes and superinfection exclusion [38], modulating pathogen aggressiveness. Thus, the extensive description of prophages presented here is the first step to uncovering their impact on *Erwinia* evolution and ecology.

Comprehensive genomic analysis pointed out that prophages are common in bacterial genomes, especially in pathogens from the *Enterobacterales* order [39]. Indeed, putative prophage sequences are pervasive in the *Erwinia* spp., indicating that they could be relevant to bacterial evolution and environmental fitness.

All *Erwinia* spp. genomes had signatures of prophage-like sequences, indicating that every strain evaluated in this study was infected by lysogenic phages during their evolutionary history or vertically inherited prophage genes. Despite this, the number and completeness of the putative prophage sequences highly depended on the *Erwinia* species. While *E. amylovora* and *E. gerundensis* had a higher proportion of defective sequences (incomplete and questionable), *E. tracheiphila* and *E. rhapontici* were enriched in putative functional prophages (intact sequences). It is important to emphasize that we used only in silico analyses to describe the occurrence of prophages in *Erwinia* spp., which is usually limited in detecting prophages with genes and genomic structures similar to previously described phages. Therefore, experimental analyses of prophage induction may reveal new sequences not detected by computational tools.

An in vitro screening for *Erwinia* phages from *Podoviridae* and *Myoviridae* families pointed out that lysogeny is rare in *E. amylovora* since any isolate had evidence of prophages using qPCR, as well as the absence of spontaneous or induced release of prophages [40]. Indeed, among the 146 assemblies analyzed, we found that only 12 genomes (8.2%) had intact sequences. The scarcity of putative inducible viral genomes in *E. amylovora* and *E. gerundensis* suggests that these species are efficient in inactivating prophages or preventing new integration events. The prophage profile in *E. amylovora* and *E. gerundensis* (Figure 2) suggests that their sequences were under a similar evolutionary trend observed for other enterobacterial prophages, in which many prophage genes are rapidly lost after genome integration, followed by a slower genetic decay of the remaining genes that could provide adaptive fitness to the cell. In such a scenario, many prophage genes in a given *Erwinia* species are possibly orthologous and derived from ancestral prophage integration events [41].

On the contrary, *E. tracheiphila*, *E. rhapontici*, and *E. persicina* apparently had different dynamics regarding prophage acquisition and evolution. These species harbored a higher number of putative prophages and a higher proportion of intact sequences. Although rapid prophage decay is a common event due to the pervasiveness of defective sequences in *Erwinia* genomes, the acquisition of new prophages seems recurrent in these species, possibly influenced by the absence of DISARM, BREX, and CRISPR-Cas anti-phage defense systems in their genomes. Thus, the domestication of new prophages tends to occur more frequently, and such horizontally acquired DNA may constitute an important evolutionary trait for these *Erwinia* species.

Furthermore, most of the isolates of a given *Erwinia* species were sampled in multiple locations. Thus, they are probably subjected to variable abiotic factors that impact phage stability and infectivity [42,43,44,45]. Therefore, the species-specific profiles of prophages observed here are likely an outcome of the genetic background and ecological niche of the *Erwinia* species and are less influenced by environmental conditions.

It is important to note that some of the *Erwinia* spp. genomic sequences were highly fragmented, mainly due to the technical limitations of genome sequencing and assembly and to the features of the sequences (e.g., GC content, large homopolymeric regions, and repetitive sequences) [46]. Some prophage-like sequences were located near contig edges and had small sizes, especially within fragmented bacterial assemblies. However, we did not exclude these sequences from the analysis since they were likely derived from prophages. From the perspective of the description of prophages in *Erwinia*, greater harm could be made if we ignore the clear signals of prophage occurrence in some bacterial genomes. We wondered if, in future releases, the prophage-finding tools could provide warnings when candidate prophages have small sizes and are near contig edges, which may hamper accurate prophage sequence delimitation and completeness estimation.

Contrasting the GC content of the hosts and phages is commonly conducted in phage characterization studies. A comprehensive genomic analysis indicated a linear relationship of GC content between phages and their hosts [30], although such a trend might be missed when analyzing a small subset of the data. We could not detect a direct correlation of GC content between prophage-like sequences and *Erwinia* genomes for the putative *Erwinia* prophages. However, such genomic features did not vary significantly between the putative prophages and their hosts (Table S10), suggesting that GC-content adjustments are in progress during prophage domestication. Furthermore, such small variations in GC content are expected since bacterial genomes naturally have GC-skew and heterogeneous distribution of nucleotides [47,48], implying that genome segments may have a GC content that is slightly different from the average value.

The sequence-based taxonomic assignment is important to group similar phages from an evolutionary perspective, which may have practical value concerning viruses’ origin, replication mechanism, and life cycle. Most classified putative *Erwinia* prophages belonged to the *Myoviridae* family, while fewer were from *Siphoviridae* and *Podoviridae* (Figure 4A,B). A similar trend was observed in previous in vitro screenings of phages infecting *Erwinia* [40,49], detecting only phages from the *Caudoviricetes* class.

Given that the cryptic filamentous viruses (class: *Tubulavirales*) are ubiquitous in prokaryotes, we expected to find filamentous prophages in *Erwinia* genome assemblies [33]. For this purpose, we employed the pipeline “Inovirus_detector” [33]. However, no inovirus-like sequences were retrieved. Furthermore, the highest scores of the Baltimore classification provided by VPF-class indicated that all candidate prophages identified by PHASTER had genomes composed by dsDNA, which was consistent with the absence of *Inoviridae* prophages within *Erwinia* assemblies according to the Inovirus_detector tool.

Overall, the set of prophage-like sequences showed a low degree of intergenomic similarities, as evidenced by the high number of species clusters with few sequences each. However, some species clusters contained a relatively high number of putative prophages of *E. amylovora* (clusters 2, 5, 7, 8, 11. Table S6), such as cluster 5 which contained 98 defective sequences, each one in a specific *E. amylovora* isolate. These sequences are possibly orthologous and transferred vertically. Although most prophage-like sequences are considerably divergent, the gene-sharing network indicated that they share many genes (Figure 3), which could be due to horizontal gene transfer, including between prophages from different *Erwinia* species.

Most of the putative *Erwinia* prophages described here are not covered in databases since only three sequences had more than 70% of intergenomic similarities to known phage sequences, according to VIRIDIC (Table S6). This evidenced that only a small fraction of *Erwinia* phages is already described in databases. Thus, this work expanded the sequence space of *Erwinia*-infecting viruses.

Bacteria possess diverse anti-phage defense systems, employing many mechanisms to reduce infection by lytic or temperate phages [50]. To better understand the profile of prophages observed in *Erwinia*, we searched for DISARM, BREX, and CRISPR-Cas defense systems. We could not detect putative DISARM systems in the analyzed *Erwinia* genomes, while the BREX system was detected only in ~2.7% of the genomes, mainly in *E. pyrifoliae* isolates. However, these *Erwinia* isolates possessed multiple prophage-like sequences, including intact ones. Thus, the temperate phages that infected *E. pyrifoliae* probably evaded their BREX mechanism since these defense systems are likely functional (complete).

Finally, we investigated the occurrence and diversity of CRISPR-Cas systems in *Erwinia* spp., given their role in bacterial immunity against phages and being the only adaptive and heritable defense system known to date [51].

Most *Erwinia* assemblies (76.9%) had putative CRISPR-Cas systems, a high frequency compared to many other enterobacteria [12]. However, the pervasiveness of this defense system is biased due to the more significant proportion of *E. amylovora* isolates in the data set, all showing CRISPR-Cas system type I-E. Many other *Erwinia* species, such as *E. gerundensis*, *E. rhapontici,* and *E. tracheiphila*, did not show CRISPR-Cas system according to the CrisprCasFinder algorithm. Although varying greatly in the *Erwinia* genus, the number and type of CRISPR-Cas system are well conserved within each *Erwinia* species (the only exception was *Erwinia persicina*), suggesting that they are under strong evolutionary constraints after speciation, and their biological relevance must be analyzed in a species-wise manner.

We could not identify most of the protospacers targeted by the *Erwinia* CRISPR spacers, indicating that there is still a large amount of undescribed mobile genetic elements (MGEs) and the need for further environmental microbiome characterization. Besides this, the accumulation of sequence mutation could hamper alignments between CRISPR spacers and targets, hindering its functionality and protospacer identification.

*E. amylovora* and *E. pyrifoliae* are closely related species and show similar disease symptoms [2]. Using sequences from public databases, we found that plasmid sequences were the most identifiable targets of the CRISPR spacers from these species, indicating that these MGEs may represent a more significant threat than phages in natural environments. The colonization niche of *E. amylovora* and *E. pyrifoliae* is the aerial parts of *Rosaceae* [2], in which they are in contact with other bacteria, possibly other *Erwinia* populations [52]. Indeed, the BLASTn best hits indicated that most of the plasmids targeted by CRISPR spacers in *E. amylovora* derived from isolates of this species (Table S11).

Interestingly, the *Erwinia* tasmaniensis phage phiEt88 sequence and the plasmid pET35 from *E. tasmaniensis* ET1/99 were prevalent among the targets of *E. amylovora* and *E. pyrifoliae* CRISPR spacers. *E. tasmaniensis* is a non-phytopathogenic species [53], which might act as an antagonist of plant pathogenic *Erwinia*. Likewise, it is known that non-phytopathogenic epiphyte *Pantoea* species could be infected by *Erwinia* phages, being used as carriers to introduce phages in populations of plant-pathogenic *Erwinia* [54,55]. Owing to the prevalence of MGEs from *E. tasmaniensis* as putative targets of *E. amylovora* and *E. pyrifoliae* CRISPR spacers, we wondered if similar events might occur between these *Erwinia* species, with an intimate association and frequent MGE exchange between bacterial populations.

Furthermore, the *Erwinia* CRISPR-Cas system targeted the candidate prophages identified by PHASTER, regardless of their completeness. The vast majority of the CRISPR spacers and the target prophage-like sequences occurred in different genome assemblies (2193/2197 = 99.8%), indicating that many putative prophages are active in the environment, possibly acting as temperate phages, infecting or being repealed by *Erwinia*.

Most of the *E. amylovora* isolates had CRISPR spacers directed to the prophage-like sequences (139/146 = 95.2%), correlating well with the low relative abundance of intact sequences in this species. Such prophage sequences are regarded as “true” phages, generally possessing a complete or near-complete set of genes necessary to virus metabolism [56]. Since CRISPR-Cas systems prevent virus infection, new phages are unlikely to infect the cell. On the other hand, *E. pyrifoliae* did not show any spacer targeting the prophage-like sequences, even though all isolates had two different types of CRISPR-Cas systems with many CRISPR arrays. Since *E. pyrifoliae* assemblies harbored multiple putative prophages, we hypothesized that their CRISPR-Cas systems are somewhat inefficient in collecting spacers against these MGEs. Thus, a bias of protospacer acquisition from phages may exist depending on *Erwinia* species, possibly influenced by the impact of prophages on cell fitness or virus strategies to circumvent CRISPR-Cas defense, such as DNA modifications and protein inhibitors [57].

We could not guarantee that the CRISPR spacers were acquired specifically from the prophage sequences since viruses generally show high levels of genetic mosaicism, sharing genome segments even between distantly related phages [58]. However, owing to the restricted ecological niche of *Erwinia* and the probable lower richness of dsDNA viromes in aerial parts of plants compared to other environments, it is very likely that the CRISPR spacers are directed to the putative *Erwinia* prophages.

## 5. Conclusions

In this study, we described the occurrence and diversity of putative prophages infecting *Erwinia* spp. The genus *Erwinia* comprises relevant plant pathogens, and, thus, an overall description of prophages is an important step toward better characterizing genomic determinants of pathogenicity and environmental fitness. In this sense, our work indicated that signals of prophage-like sequences are pervasive in *Erwinia* genomes. However, the putative functional (intact) sequences were unevenly distributed within the genus, indicating that the genetic background of bacteria plays a key role in the acquisition and fate of prophages. Overall, we identified a highly diverse set of sequences; most were previously undescribed, contributing to unveiling the diversity of prophages infecting *Erwinia*. Since we could not find a prophage-like sequence occurring in different *Erwinia* species, we inferred that temperate *Erwinia* phages have a narrow host range. Likewise, the occurrence and type of CRISPR-Cas systems were species-specific. On the other hand, BREX and DISARM systems were rare or even absent in the analyzed *Erwinia* spp. The presence or absence of these defense systems did not necessarily reflect the occurrence of prophages in *Erwinia* genomes, indicating that other genetic traits or ecological factors influence *Erwinia* infection by phages. As more bacterial genomes and metagenomes are sequenced, a deeper sampling of phage diversity is becoming available, which could provide genomic data to support why ssDNA phages, e.g., inoviruses and microviruses, are rare or even absent in *Erwinia*. The knowledge generated can increase our understanding of phage–*Erwinia* interaction and phage applicability, such as in controlling pathogenic *Erwinia* populations.

## Data Availability

Data is free available at FigShare https://doi.org/10.6084/m9.figshare.21401790.

## Supplementary Materials

The following supporting information can be downloaded at: https://www.mdpi.com/article/10.3390/pathogens12010044/s1. Figure S1: Statistics of the *Erwinia* spp. data set used in this study; Figure S2: Comparison of the GC-content of the putative prophages and their hosts; File S1: Fasta sequences of the putative *Erwinia* prophages detected using the PHASTER tool. The file is freely available at FigShare https://doi.org/10.6084/m9.figshare.21401790; Table S1: Description of defective *Erwinia* prophages sequences near bacterial contig edges and having less than 20 kb; Table S2: Distribution of *Erwinia* prophages in each *Erwinia* spp. genome assembly; Table S3: The genome_by_genome_overview.csv output file from vConTACT2; Table S4: Taxonomic assignment of prophages (PHASTER) by vConTACT2 and VPF-class; Table S5: The genome_by_genome_overview.csv output file from vConTACT2 showing only the 46 unclassified intact *Erwinia* prophage sequences using a combination of vConTACT2 and VPF-class; Table S6: The clusters.csv output file from VIRIDIC; Table S7: Distribution of CRISPR-Cas systems in *Erwinia* spp. Genomes predicted using CRISPRCasFinder v.4.2.20; Table S8: CRISPR spacers targeting de prophages; Table S9: Putative BREX type 1 defense system detected in *Erwinia* spp. genome assemblies; Table S10: Genomic features of *Erwinia* species and prophages; Table S11: CRISPR spacers showing significant BLASTn hits to plasmid and viruses sequences from databases.
