# Supplementary material for: Genomic Analysis Unveils the Pervasiveness and Diversity of Prophages Infecting Erwinia Species
Source: Pathogens. 2022 Dec 27;12(1):44. doi: 10.3390/pathogens12010044 (PMC9866893; doi:10.3390/pathogens12010044)
Supplement: Supplementary file 1 [file pathogens-12-00044-s001.zip › Figure S1.pdf]

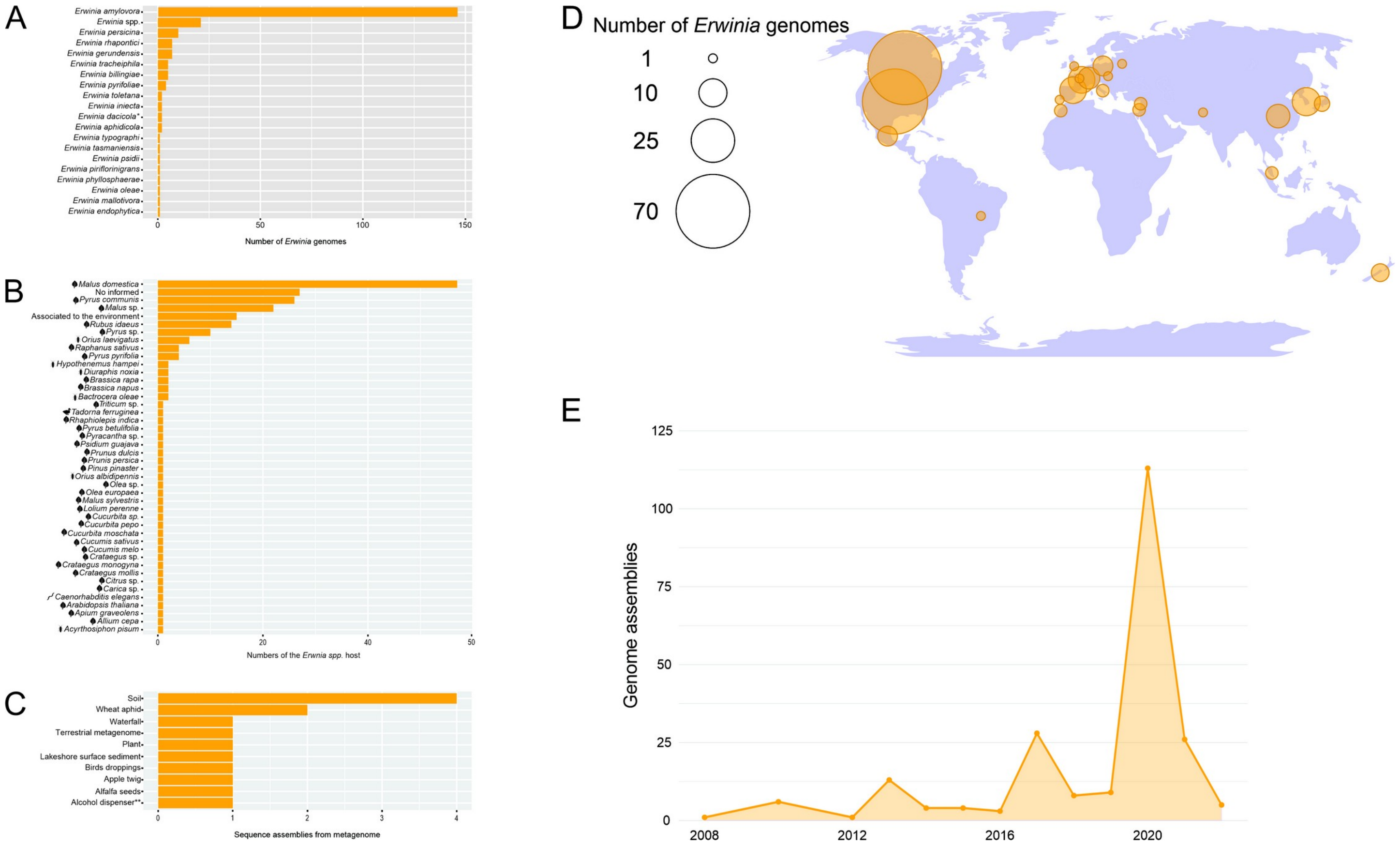

Figure S1. Statistics of the *Erwinia* spp. data set used in this study. (A) Distribution of genome assemblies by species. (B) Distribution of *Erwinia* hosts. (C) Number of *Erwinia* spp. genomes obtained from environmental samples. (D) Distribution *Erwinia* spp. genomes by geographical location. (E) Number of genome assemblies deposited in GenBank by year.
