# Supplementary material for: Genomic Analysis Unveils the Pervasiveness and Diversity of Prophages Infecting Erwinia Species
Source: Pathogens. 2022 Dec 27;12(1):44. doi: 10.3390/pathogens12010044 (PMC9866893; doi:10.3390/pathogens12010044)
Supplement: Supplementary file 1 [file pathogens-12-00044-s001.zip › Figure S2.pdf]

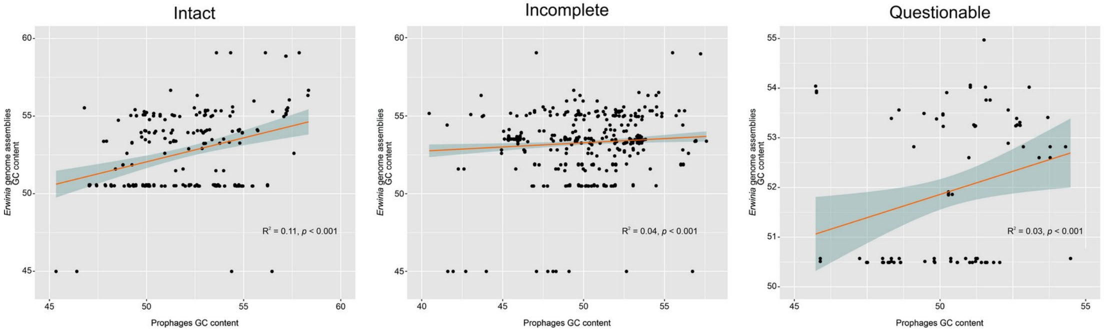

Spearman's correlation coefficient of *Erwinia* bacteria GC content and *Erwinia* prophage GC content	
Erwinia Prophage	Rho
Intact	0.11
Incomplete	0.04
Questionale	0.03
p < 0.001; Strength of correlation: Small (0.1 to 0.3), Medium (0.3 to 5), large (0.5 to 1.0) and no correlation (0.0) (-) indicates a negative correlation and (+) a positive correlation.	

Figure S2. Comparison of the GC content of prophages and their hosts. The GC content showed weak correlation (Spearman's coefficient) and the regression models poorly explained the observed data (low R-squared values).
